# Immersive virtual reality as analgesia for women during hysterosalpingography: study protocol for a randomized controlled trial

**DOI:** 10.1186/s13063-019-4023-y

**Published:** 2020-01-20

**Authors:** Yi-Ling Wang, Hai-Xiang Gao, Jin-She Wang, Jing-Hui Wang, Lei Bo, Ting-Ting Zhang, Ya-Liang Dai, Lu-Lu Gao, Qiang Liu, Jun-Jun Zhang, Ju-Min Cai, Jian-Qiang Yu, Yu-Xiang Li

**Affiliations:** 1 School of Nursing, Ningxia Medical University, 1160 Sheng Li Street, Yinchuan, 750004 China; 2 Nursing Department, The First People’s Hospital of Yinchuan, 2 Li Qun Street, Yinchuan, 750004 China; 3 School of Science, Ningxia Medical University, 1160 Sheng Li Street, Yinchuan, 750004 China; 4 Radiology Department, Yinchuan Women and Children Healthcare Hospital, 56 Wen Hua Street, Yinchuan, 750004 China; 5 Foreign Language Teaching Department, Ningxia Medical University, 1160 Sheng Li Street, Yinchuan, 750004 China; 6 School of Public Health and Management, Ningxia Medical University, 1160 Sheng Li Street, Yinchuan, 750004 China; 7 School of Preclinical Medical Sciences, Ningxia Medical University, 1160 Sheng Li Street, Yinchuan, 750004 China; 8 Department of Pharmacology, Pharmaceutical Institute of Ningxia Medical University, 1160 Sheng Li Street, Yinchuan, 750004 China

**Keywords:** Hysterosalpingography, Analgesia, Virtual reality, Pilot study

## Abstract

**Background:**

Hysterosalpingography (HSG) is an accepted radiologic diagnostic modality for initial infertility workup, and is generally considered uncomfortable and painful. However, the management of pain related to HSG remains inefficient. As an emerging nonpharmacologic and noninvasive pain control strategy, virtual reality (VR) distraction has been successfully used in areas such as burns, blunt force trauma, hospital-based needle procedures, dental/periodontal procedures, and urological endoscopy patients. This study aims to evaluate the analgesic effect of VR during HSG.

**Methods/design:**

A single-center, parallel-group, randomized controlled trial will be carried out in the Radiology Department of Yinchuan Women and Children Healthcare Hospital, Yinchuan. A total of 200 participants who are scheduled for HSG will be enrolled in this study. The participants will be randomized (1:1) into two groups: a VR group and a blank control group. The VR group will receive routine care plus immersive VR intervention and the blank control group will receive routine care. Outcomes will be monitored at baseline, immediately after HSG and 15 min after HSG for each group. The primary outcome is the worst pain score during HSG by a visual analog scale (VAS). The secondary outcomes include: affective pain, cognitive pain, and anxiety during the HSG procedure; worst pain within 15 min after HSG; patient satisfaction and acceptance with pain management; physiological parameters; adverse effects; HSG results; and immersion perception score of the VR system (for the VR condition only).

**Discussion:**

This study will focus on exploring a simply operated, noninvasive and low-cost analgesia during the HSG procedure. The results of this trial will provide data on the feasibility and safety of VR distraction therapy during HSG.

**Trial registration:**

Chinese Clinical Trial Register, ChiCTR1900021342. Registered on 16 February 2019.

## Background

Infertility is clinically defined as 1 year of attempted conception that has not resulted in pregnancy; it is one of the most prevalent chronic health defects impacting young couples [1]. Approximately 6.6–26.4% [2] of couples of reproductive age are confronted with infertility in the industrialized nations. The primary physiological factors leading to female infertility are fallopian tube abnormalities, accounting for about 30–40% [3]. Hysterosalpingography (HSG) is an accepted radiologic diagnostic modality for initial infertility workup which can assess the patency of the fallopian tube and the womb cavity situation [4].

HSG is a minimally invasive gynecological procedure performed in the outpatient department which is generally considered to be uncomfortable and painful. It has been reported that 85% of women suffer operation pain, with half complaining of moderate to severe pain [5, 6]. Currently, it is generally believed that the pain related to HSG is mainly caused by cervical instrumentation, uterine instillation of contrast material, peritoneal irritation secondary to contrast material spill, or delayed postoperative pain due to prostaglandin release [7]. The uncomfortable and painful experience of the operation can lead to a high degree of stress, poor compliance with HSG procedures, and an unwillingness to undertake other similar diagnostic investigations [8]. It is very important to find an ideal analgesic method that can provide adequate pain control in the absence of significant adverse reactions.

Various interventions have been assessed to decrease pain and increase patient compliance during HSG, including paracetamol [9], nonsteroidal anti-inflammatory drugs such as fenoprofen and naproxen, opioid analgesics such as tramadol [10], intracervical and intrauterine topical anesthetic [5, 6, 11, 12], and a paracervical block utilizing lignocaine. Although studies have shown that these analgesics have certain effects on HSG pain relief, Ahmad et al. [7] conducted a systematic review and meta-analysis of related randomized controlled trials and reported that there is no consensus concerning the optimal analgesic or timing of its administration. Furthermore, in our institution, no analgesic guideline exists to direct the gynecologists to prescribe analgesics and no effort has been made to enhance gynecologist use of analgesia. As a result, patients receive no analgesics during HSG as routine care in our institution.

Nonpharmaceutical therapy, including music therapy, hypnosis, massage and distraction, can significantly improve the patient experience while undergoing painful medical procedures [13, 14]. Researchers have recently proposed that immersive virtual reality (VR) can be applied as an unusually powerful psychological therapy for acute pain relief [15]. The essence of immersive VR is the user’s illusion of going inside the three-dimensional computer-generated world through a head-mounted device, as if the virtual world is a place the user is visiting [16]. Hoffman et al. proved that VR can relieve pain caused by blunt instrument injury [17] and wound debridement as well as dressing burn injuries [15]. Furman et al. [18] conducted clinical trials on dental surgery patients, showing that VR effectively alleviates pain and fear of dental surgery.

To date, and to the best of our knowledge, no study has examined the effect of VR intervention on women undergoing HSG. This clinical randomized controlled trial aims to evaluate whether VR intervention, when compared with a blank control group, could decrease pain and to evaluate the effects of pain relief on the ease of performing HSG for infertility diagnosis.

## Methods/design

### Study design

The primary objective of this single-center, parallel-group, randomized controlled trial is to find out the clinical analgesic effect of VR intervention in patients during HSG compared with a blank control group. The secondary objectives of this trial include comparing the anxiety level during HSG between the two groups, evaluating the safety of clinical use of VR by recording adverse effects and physiological indexes, assessing patient acceptance for the nonpharmaceutical analgesia therapy and investigating the satisfaction of patients with VR.

**Fig. 1:**
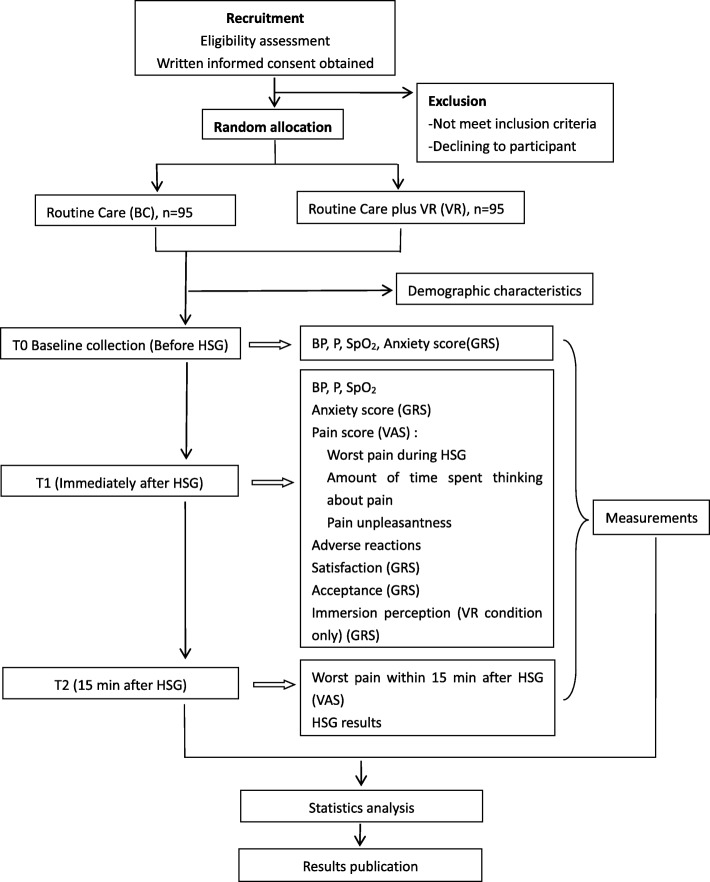
Study design framework. BC blank control, BP blood pressure, GRS graphic rating scale, HSG hysterosalpingography, P pulse, SpO_2_ oxygen saturation, VAS visual analog scale, VR virtual reality

**Fig. 2:**
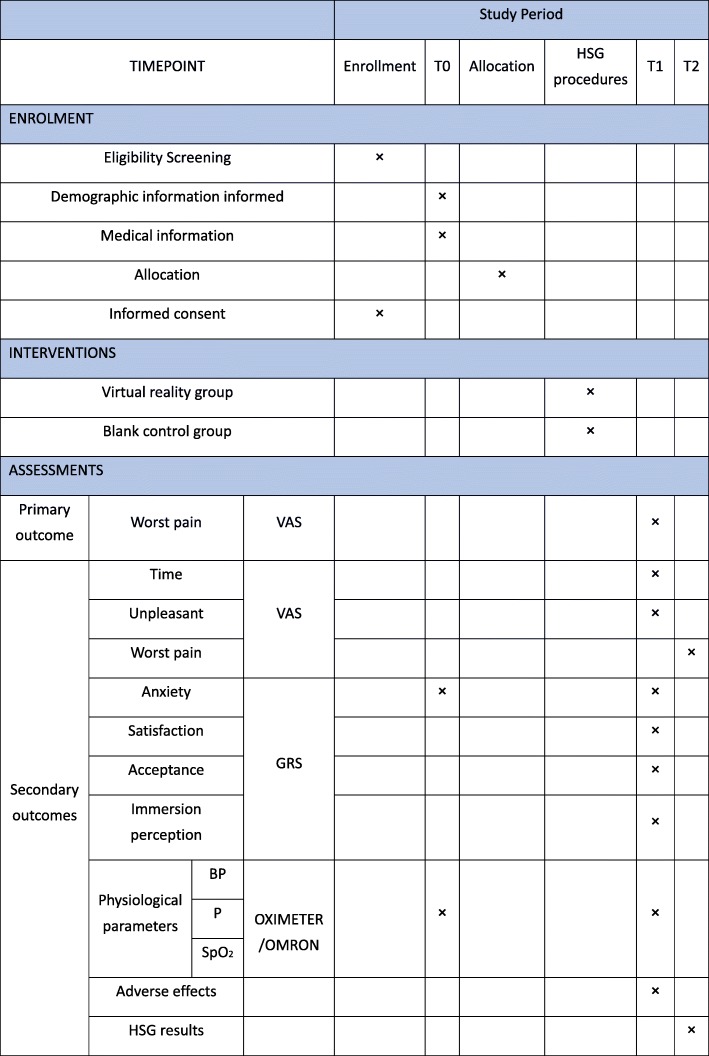
SPIRIT schedule of enrolment, interventions, and assessments. BP blood pressure, GRS graphic rating scale, HSG hysterosalpingography, P pulse, SpO_2_ oxygen saturation, T0 baseline, T1 immediately after HSG, T2 15 min after HSG, VAS visual analog scale

The trial has been approved by the Institutional Ethics Committee of Ningxia Medical University (reference no. 2018–233). This randomized controlled trial has been registered in the Chinese Clinical Trial Registry (ChiCTR1900021342). The whole study design is illustrated in Fig. 1. Additionally, a Standard Protocol Items: Recommendations for Interventional Trials (SPIRIT) figure for the schedule of enrolment, interventions, and assessments is presented in Fig. 2. We will conduct the protocol of this trial according to the SPIRIT guidelines (see Additional file 1).

### Study setting

This study will be conducted in the HSG room of the Radiology Department of Yinchuan Women and Children Healthcare Hospital in Ningxia province. This institution is an urban, public, tertiary-care teaching hospital, specializing in maternal care and childcare in Ningxia Hui Autonomous Region located in Northwest China. Annually 50,000 patients undergo treatment at the fertility clinic and approximately 1000 HSG are performed every year in this institution. It is therefore feasible to reach the target sample size in this selected clinical setting. We expect to complete recruitment within 10 months.

### Study participants

Women will be recruited into the study according to the following inclusion criteria: 1) indications for HSG including infertility, recurrent miscarriage, and other miscellaneous reasons [5]; 2) aged 20–45 years; 3) willing to participate in the study and sign informed consent; and 4) no signs of pain before HSG.

The exclusion criteria are: 1) use of antidepressants or sedatives; 2) presence of psychiatric disorders; 3) recent receipt of analgesics within 6 h; 4) allergy to contrast medium or iodine; 5) a recent history of acute pelvic inflammatory disease; 6) visual acuity <1.0 or hearing abnormity; 7) susceptible to motion sickness; 8) operator difficulty due to cervical adhesion or congenital variation, or completing the examination after multiple operations.

In the Radiology Department, the HSG procedure will be carried out on each weekday by two gynecology assistants (both female who have worked in the HSG room for >10 years). The results of the HSG will be analyzed by a Chief Radiologist (who has worked in the HSG room for >10 years). These two gynecology assistants will receive training from the protocol conceiver.

### Recruitment

Recruitment will take place in the Radiology Department of Yinchuan Women and Children Healthcare Hospital. Participants for the study will be recruited through WeChat advertising and posters in the infertility clinic. After patients arrive at the Radiology Department they will be screened with a free preliminary physical examination and a check against the inclusion/exclusion criteria. The potential participants will receive information about the aims, benefits, and latent risks of the study from the protocol designer. If the patients wish to participate, they will be asked to sign an informed consent form that they agree to participate in the study and are willing to let the protocol designer publicize the research results, including pictures. Participants are entitled to withdraw from the study at any time and this will not prejudice their subsequent treatment. The whole process of recruitment follows the principle of respect for autonomy.

### Randomization, allocation concealment and blinding

A simple random sampling method will be used in our study. Patients who are scheduled for HSG and meet the eligibility criteria will be randomly allocated into either the blank control group or the VR group at a 1:1 ratio. The randomization sequences will be generated in advance with a computerized random number generator (random.org). This is performed by a statistical expert who is not involved in this trial. A sealed opaque envelope will be used to store the randomization sequences of all allocations. The random number table will be kept confidential by the full-time secretary of this project. Baseline data will be recorded as close to immediately before the HSG procedure as possible. After baseline data collection, the allocation will be revealed. Blinding is not feasible.

### Virtual reality prototype

**Fig. 3:**
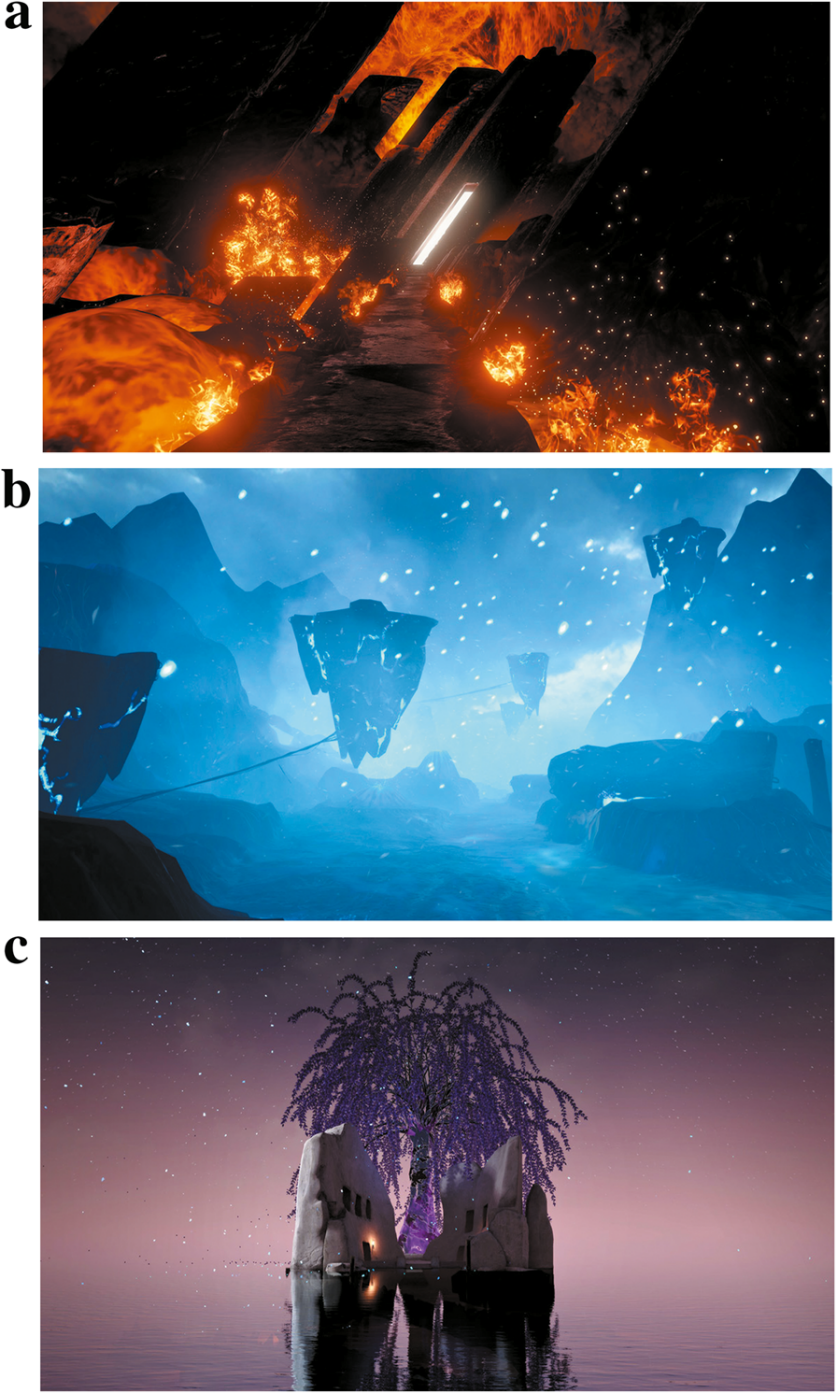
Screenshots of the virtual scenes provided for participants in the virtual reality group. **a** The land of magma. **b** The frozen ground. **c** A flowering tree in the violet world

The immersive VR prototype has been constructed by combining commercial, advanced, off-the-shelf components. The prototype consists of an HTC VIVE VR system and a VIVE-ready computer with a head-mounted display helmet with noise-reducing headphones, two wireless controllers and two base stations included with the HTC VIVE VR system. The head-mounted display helmet will be linked to a desktop computer (Dell OptiPlex 7050, Windows 10 64-bit operating system, Intel® Core™ i7-7700 processor, 3.60 GHz central processing unit, 8.0 GB installed random-access memory, Intel® HD Graphics 630 video card) with Steam VR software. The VR environment comes from Zamer which is a software platform to distribute VR content (www.zamertech.com). Under VR treatment, participants can travel on a canoe slowly through three magic virtual scenes (Fig. 3). Music in the virtual scenes is an adapted opera. The immersive VR prototype developed by HTC will be adapted to the HSG room in the Radiology Department. The main researchers will be trained in proficiency with VR intervention in the Ningxia Medical University Innovation and Entrepreneurship Park of Health and Medicine.

### Hysterosalpingography procedures

HSG will be scheduled by gynecologists between 3 and 7 days after the end of menstruation. Any history of iodine allergy and unfavorable pregnancy will be obtained. The colloidal gold test paper will be used to exclude early pregnancy. In the preoperative waiting room, patients arranged for HSG will receive routine care without any analgesia provided. Atropine (0.5 mg) will be administered intramuscularly approximately 30 min before HSG to prevent fallopian tube spasm which could lead to false obstruction during the imaging examination during HSG.

After placing the patients in the lithotomy position on the fluoroscopic table, the gynecology assistants help them to flex and separate the thighs to fully expose the vulva and then perform vaginal irrigation for local cleaning with antiseptic 1% chlorhexidine solution. After disinfecting the vulva with a standard 2000 mg/L iodophor solution, a sterile bivalve speculum is introduced into the vagina to visualize the uterine cervix. A 4-mm sterile uterus radiography balloon catheter is inserted through the cervical canal and a tenaculum used to hold the cervix only if it is difficult to insert. The gynecology assistant injects 2 mL air into the aerocyst in the uterus radiography balloon catheter. After filling the balloon, it is gently pulled back to fix the catheter in the internal cervical orifice to prevent contrast leakage. A water-based iodine contrast medium (Omnipaque 300; GE Healthcare, Shanghai, China) in volumes of 5–15 ml is instilled through the uterus radiography catheter with fluoroscopic x-ray images obtained intermittently to assess the uterus and fallopian tubes. The whole procedure lasts about 20 min. At the end of the operation, the cannula and instruments are removed and the patient remains under observation for 15 min in the Radiology Department before discharge.

### Interventions

**Fig. 4:**
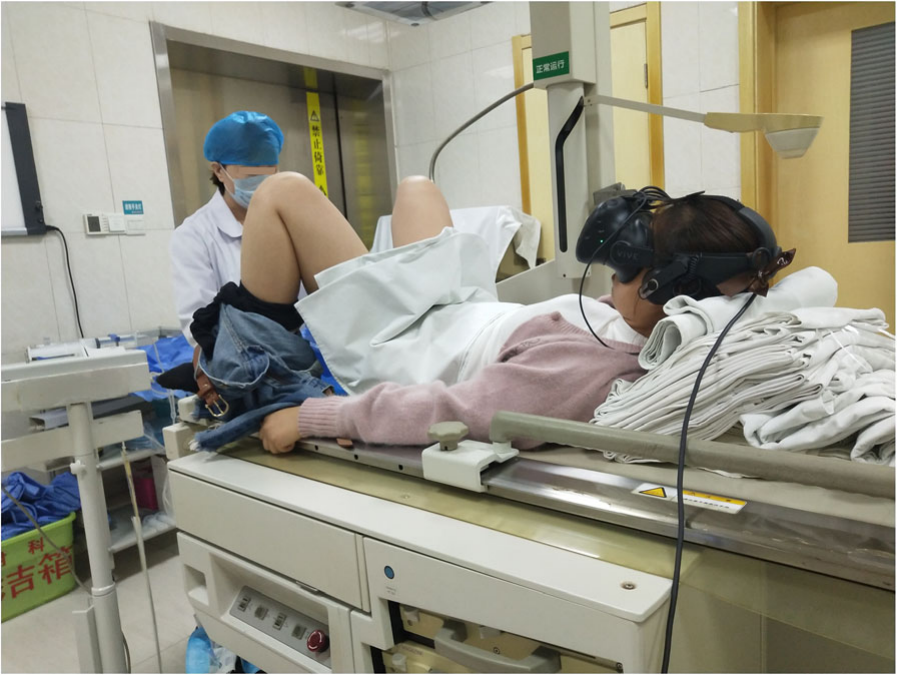
A patient undergoing a hysterosalpingography procedure on the fluoroscopic table ‘enters’ the immersive virtual world

In the blank control arm, gynecology assistants will perform the whole procedure as they usually would. In the VR group, the immersive VR system will be provided (Fig. 4). After placing the patient in the lithotomy position, the trained investigator will help the patient wear the specific helmet and teach them how to interact with the virtual world through gaze-based tracking and the wireless controllers. The gynecology assistant will carry out the relevant operation 2 min later. VR distraction will be applied during the whole procedure. Patients in the VR group will be asked how many virtual scenes they ‘enter’ to monitor adherence to intervention protocols.

### Measurements

Demographic data (age, nationality, education, occupation) and physiological parameters, including pulse rate, blood pressure and oxygen saturation, will be recorded by the investigator. In order to evaluate gynecologic and pelvic pain-associated histories, patients will be examined and their relevant medical history will be collected (duration of infertility, primary or secondary infertility, vaginal or cesarean delivery, prevalence of dysmenorrhea and dyspareunia, history of dilatation and curettage, HSG, and abdominal surgery or infection). Other indexes regarding pain score, baseline anxiety level, anxiety level during HSG, adverse effects, patient satisfaction and acceptance of pain management, as well as the immersion perception of the VR system, will be recorded by the researcher.

Cognition and emotion are important factors known to regulate pain perception in clinical settings and laboratories [19]. Sensory and affective pains are separate dimensions of the pain experience and can be measured respectively [20]. Thus, like other clinical VR studies [15, 21], subjects will be asked to complete three self-assessment pain outcomes in this study: the sensory pain dimension (worst pain), the cognitive pain dimension (amount of time spent thinking about pain) and the affective pain dimension (pain unpleasantness). Participants will respond to questions about worst pain, amount of time spent thinking about pain, and unpleasantness they experience during HSG by using a visual analog scale (VAS) (range = 0 to 10). VAS is a widely used self-report instrument in younger adults [22]. The extremes of the 10-cm line VAS are “no pain at all/none of the time/not unpleasant at all” and “worst pain possible/all of the time/excruciatingly unpleasant” [23–25]. Moreover, baseline anxiety level, anxiety level during HSG, patient satisfaction and acceptance of pain management, and the immersion perception of the VR system will be measured using a graphic rating scale (GRS) labeled from 0 to 10 labeled. Gracely et al. [20] have proved ratio scale measures such as the GRS to be highly reliable.

Any observed adverse effects will be carefully evaluated and recorded by the investigators. The expected adverse effects include gastrointestinal adverse reactions (nausea, vomiting), adverse effects on the cardiovascular system (arterial hypotension and bradycardia) and any other discomfort (pallor, sudation, palpitation, dizziness, syncope). If any adverse effects take place, health care givers will administer corresponding treatment or nursing care. All severe adverse reactions and solutions during the procedure will also be noted. HSG results (both tubes patent, one or both fallopian tubes occluded and/or impaired patency) should be recorded and compared. The physiological indexes will be monitored in terms of treatment safety with an electronic manometer (OMRON, HEM-7120) and oximeter (PC-60B).

Participants’ demographic data, relevant medical history and baseline anxiety level, as well as pulse rate, digital monitoring of oxygen saturation and noninvasive monitoring of blood pressure, will be collected at baseline (T0) before the procedure. Patients will complete a series of assessment including three pain dimensions, physiological parameters (including pulse, blood pressure and oxygen saturation), anxiety level during HSG, satisfaction and acceptance of pain management and adverse reactions along with the immersion perception of the VR system (for the VR group only) immediately after the procedure (T1). HSG results and worst pain will be recorded 15 min (T2) after the procedure. To promote participant retention, every woman who completes all data collections will receive a free overall analysis of the HSG results by an experienced gynecologist.

### Outcome measures

#### Primary outcome measure

The primary outcome measure will be the worst pain score experienced during HSG (by VAS). It will be collected by asking patients to rate “Your WORST pain intensity during HSG?”, ranging from no pain at all to worst pain possible.

#### Secondary outcome measures

Secondary outcome measures include: 1) pain score of the cognitive component (by VAS), collected by asking patients to rate “How much time did you spend thinking about your pain during HSG?”, ranging from none of the time to all of the time; 2) pain score of the affective component (by VAS), collected by asking patients to rate “How UNPLEASANT was your pain during HSG?”, ranging from not unpleasant at all to excruciatingly unpleasant; 3) worst pain score within 15 min after HSG (by VAS); 4) anxiety score before/during HSG (by GRS), collected by asking patients to rate “How much ANXIETY did you have before/during HSG?”, ranging from none at all to extremely anxious; 5) patient satisfaction score with pain management (by GRS), collected by answering the question: “How satisfied were you with the holistic pain management during HSG?”, ranging from not satisfied at all to completely satisfied; 6) patient acceptance score of pain management (by GRS), collected by answering the question: “How receptive were you to overall pain management during HSG?”, ranging from not receptive at all to completely receptive; 7) immersion perception score of the VR system (by GRS), collected by answering the question: “To what extent did you feel like that you went into the virtual reality environment while experiencing VR?”, ranging from “I did not feel like I went into the virtual reality environment at all” to “I went completely into the virtual reality environment” (for the VR group only); 8) physiological parameters; 9) adverse effects; and 10) HSG results.

### Data monitoring

A Data Monitoring Committee, which is independent of any competing interests, will be established immediately after the project launch. Members consist of one pain management specialist, one chief radiologist, two gynecology assistants working in the Radiology Department, and a senior academic statistician who will serve as the committee’s chair. Regular Data Monitoring Committee meetings will be held to discuss project issues, modify the protocol and monitor the progress of the study. No audit or interim analyses are planned.

### Data storage

Collected paper-based materials will be stored in locked filing cabinets in a locked office in Ningxia Medical University. All data will be collected in password-protected digital files on a password-protected computer in a locked office. Data entry will be performed continually throughout the study using the double-entry method. When the investigator inputs the information into the computer, each participant will be given a unique numeric code (ID number). As a result, the data acquired from patients are individually identifiable by the research team members only. Only deidentified information will be presented or published. To guarantee data quality, an independent clinical research assistant will verify the completeness and accuracy of the data by review of medical records once a month. Authorship will follow guidelines recommended by the International Committee of Medical Journal Editors (ICMJE).

### Sample size determination

The worst pain severity as the primary end-point measure is employed to estimate sample size in the current research according to previous studies on physical therapy for pediatric burn injury pain and dentalgia pain [19, 24]. According to our previous study on breakthrough pain [26], we used a pilot trial to determine the sample size. Fourteen subjects recruited in May 2019 were invited to the pilot trial. Seven of them were included in the VR intervention group during HSG, and worst pain scores of two parallel groups were recorded at T1. The sample size calculation formula is based on a two-group, parallel controlled design:

$$ \mathrm{n}=\frac{\left({{\mathrm{q}}_1}^{-1}+{q_2}^{-1}\right){\left({t}_{\alpha /2}+{t}_{\beta}\right)}^2{\sigma_c}^2}{\delta^2} $$

where *n* represents the total sample size of the whole study, *σ*_c_ represents the standard deviation, *δ* represents the between-group difference with clinical significance, and q1 and q2 are the sample size ratio of the two groups (q1 = q2 = 0.5). According to the preliminary test, the worst pain score experienced during HSG reported by all blank control group participants (*n* = 7) revealed a mean (standard deviation (SD)) of 6.50 (2.26) out of 10. Participants (*n* = 7) in VR group reported a mean (SD) of 5.61 (0.93). The standard deviation of the whole sample (*n* = 14) was 1.72. At an *α* value of 0.05, a power of 90% (β = 0.10), and given a two-tailed test, we obtained *σ*_c_ = 1.72, *δ* =  ∣ *μ*_1_ − *μ*_2_∣ = 0.89. These data were used to calculate the value of the overall parameter to enter into the formula, and an *n* value of 157.02 was obtained. Thus, the rounded total sample size of the two groups was 158 (79 participants in each group). Allowing for a 20% dropout rate and withdrawals before study completion, we decided to enroll a total of 190 participants (95 participants in each group).

### Statistical analysis

In this study, all data will be collected by a specialized researcher. A blinded statistician who is not involved in this trial will perform statistical analysis using SPSS version 22.0 (Chicago, IL, USA). A complete statistical analysis plan will be generated in advance by the trial statistician. All randomized participants should be included in the analysis, and missing data will be handled using multiple imputation. Data will be analyzed initially on an intention-to-treat basis. For comparing the primary outcome between the two parallel groups, additional per-protocol analysis will be performed, and reasons for any protocol violations will be reported. We will consider all *P* values <0.05 to be statistically significant.

Descriptive statistics will be used to describe the demographic features by means (SD), medians (interquartile ranges; minimum to maximum), and proportions (exact binomial 95% confidence intervals). A *t* test will be applied to determine the differences between the primary outcome. Secondary outcomes such as the amount of time spent thinking about pain, pain unpleasantness, worst pain within 15 min after HSG, anxiety, satisfaction, and acceptance with pain management will use *t* tests or Mann–Whitney tests. Chi-squared tests or Fisher’s exact test will be used for HSG results and adverse reactions. When comparing the physiological parameters, the repeated measures analysis of variance will be used. Pearson correlation analysis will be used to analyze the relationship between immersion perception score of the VR system and the three pain dimensions.

## Discussion

HSG is routinely performed on an outpatient basis in most settings as an initial diagnostic tool for assessing infertility in women. The mechanism of pain related to HSG is complex, and there are many influencing factors. Although the main causes of HSG pain are not known, researchers have considered different methods to reduce pain during the HSG procedure, such as replacing the metal cannula with a balloon catheter and replacing oil-based media with iodinated hydro-soluble contrast media [27, 28]. Various analgesic agents have been assessed during HSG such as topical analgesics and nonsteroidal anti-inflammatory drugs. However, there is no consensus that any analgesic method is superior to others. It is well known that the intravenous administration of opioids is valid for pain relief. In a previous study, Cengiz et al. [29] explored the efficacy of the analgesic action of remifentanil compared with placebo during HSG procedure. VAS scores were recorded to assess pain during HSG in two groups. They found that women receiving remifentanil reported significantly less pain than women in a placebo group (1.25 ± 1.31 versus 4.78 ± 1.70, *P* < 0.01). Although none of the study participants reported side effects, the risk of inhibition of respiration after opioid administration necessitates continuous monitoring. The ideal analgesic for outpatient operations would have the characteristic of providing adequate pain control with an absence of significant adverse effects. For safety reasons, opioids are effective for pain management during HSG but cannot be used in outpatient settings.

With a deeper recognition of pain mechanisms, multimodal analgesia will tend to be used for pain management. Compared to traditional treatment, multimodal analgesia is a multidisciplinary approach to pain management that can maximize the positive effects of the treatment while decreasing the associated adverse effects [30]. The efficacy and adverse reactions of analgesic therapy are dominant determinants of patient satisfaction.

VR distraction is an emerging nonpharmacologic and noninvasive analgesic. It is commonly used for procedural pain management with few adverse effects. Pain perception requires attention [31, 32]. The logic for how VR works is that a substantial amount of the user’s limited controlled attentional resources is occupied with interacting with VR. Thus, when in VR the user has less attention available to process signals from pain receptors [15]. As a result, patients with burn injury report less pain during VR, less time thinking about pain while in VR, and sometimes report having more fun during the wound care procedure while in VR compared with no VR [33, 34]. Other studies exploring the efficacy of VR distraction for blunt force trauma [17], hospital-based needle procedures [35], dental/periodontal procedures [16, 18], and urological endoscopy patients [36] show that VR is efficacious in decreasing pain and adverse effects.

In the above studies, VR was used as an adjuvant to pharmacologic analgesic. We realize that it is necessary to provide a direct comparison between VR intervention and a blank control. Patients can benefit if the simply operated, noninvasive and low-cost VR application is proven to be effective during HSG procedure. Furthermore, the use of VR can have the potential to improve the safety of invasive outpatient checkups and to benefit patients from a cost-effectiveness perspective. Therefore, our study will evaluate the reliability of VR by providing data regarding the efficacy and safety of VR application in comparison with a blank control in a randomized controlled trial.

### Limitations

There are several limitations to this trial. The treatment condition will not be blinded, which may cause the participants to under-report their VAS pain scores after the VR intervention. Additionally, the questionnaire data from the participants on their three pain dimension scores, anxiety and side effects during HSG will not be collected during the procedure. Relying on recalled pain is not accurately reflecting the degree of pain in the prevailing circumstance. Nevertheless, during the distraction activity, asking patients questions will impact the efficacy of the intervention. The study will be conducted in one setting only. A multicenter study would be more representative and, in the future, we will perform a multicenter study.

### Trial status

At the time of manuscript submission recruitment for this study is ongoing. It is anticipated that the enrollment will be completed by the end of February 2020. At present, the trial has already recruited 22 participants.

## Supplementary information

**Additional file 1.** SPIRIT 2013 checklist: recommended items to address in a clinical trial protocol and related documents.

## Data Availability

After this study is completed, the final dataset and statistical codes will be accessible from the corresponding authors on reasonable request, except for patients’ personal information. The results will be published in peer-reviewed journals. Findings will be shared with the academic community, policymakers, and the general public.

## Abbreviations

GRS: Graphic rating scale
HSG: Hysterosalpingography
VAS: Visual analog scale
VR: Virtual reality
